# The Active Soil Layer of Thawing Permafrost Is an Emergent Source for Organic Substances of Concern to Water Resources

**DOI:** 10.1021/acs.estlett.5c00275

**Published:** 2025-04-21

**Authors:** Min Han, Biao Jin, Hans Peter H. Arp

**Affiliations:** † State Key Laboratory of Advanced Environmental Technology, Guangzhou Institute of Geochemistry, Chinese Academy of Sciences, Guangzhou, 510640, China; ‡ University of Chinese Academy of Sciences, Beijing 10069, China; § Guangdong Provincial Key Laboratory of Environmental Protection and Resources Utilization, Guangzhou 510640, China; ∥ Norwegian Geotechnical Institute (NGI), P.O. Box 3930, Ullevaal Stadion, N-0806 Oslo, Norway; ⊥ Norwegian University of Science and Technology (NTNU), NO-7491 Trondheim, Norway

**Keywords:** Permafrost, Water quality, Machine learning, Chemicals, Climate change

## Abstract

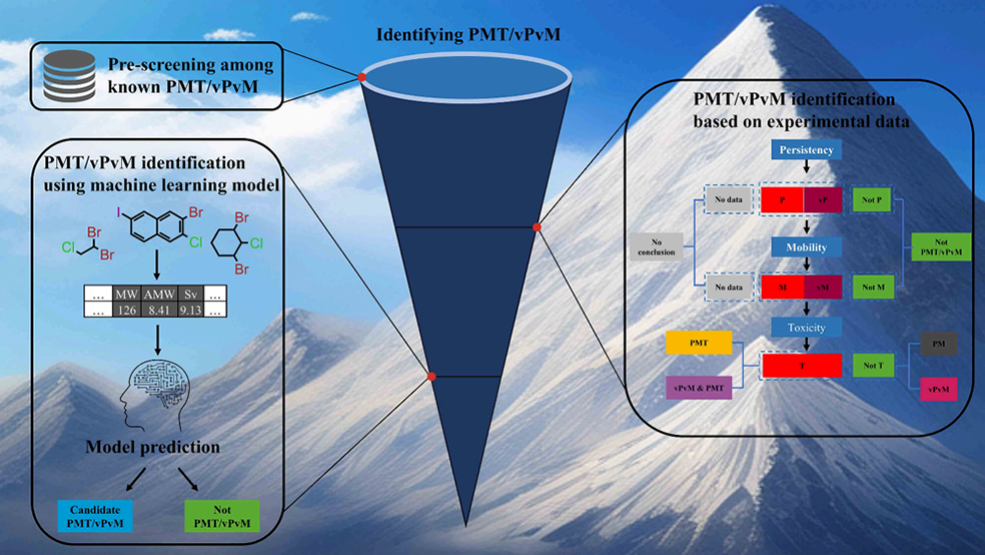

The Tibetan Plateau and surrounding area are an important source of freshwater for approximately two billion people. Climate change has aggregated permafrost degradation in the Tibetan Plateau over the last few decades, mobilizing organic substances sequestrated in the permafrost. Of particular concern are the mobilized organic substances that would be considered persistent, mobile, and toxic (PMT) or very persistent and very mobile (vPvM). These PMT and vPvM substances would persist and be widespread in the downstream water distribution system, potentially threatening drinking water sources and groundwater quality. Our study evaluated and identified PMT and vPvM substances among 21 currently available literature reports that reported detected organic compounds in the active soil of permafrost. Our approach combined a standard evaluation scheme and a machine learning model. We reported that 34% of these detected compounds are PMT/vPvM substances; these compounds were of either synthetic, natural, or undefined origin. The impact that further permafrost degradation will have on releasing these PMT/vPvM substances on water resources should be prioritized.

## 1 Introduction

The Tibetan Plateau has the most expansive alpine permafrost globally.^1−3^ This unique feature makes it particularly suitable for extensive chemical storage over prolonged periods from atmospheric deposition and local emissions.^4−6^ Over the course of millennia, a myriad of natural processes has collectively led to the accumulation of an extensive range of compounds currently sequestered within permafrost, including a recent accumulation of anthropogenic substances and their transformation products.^4,7−9^ Zhu et al.^10^ reported that the permafrost of the Tibetan Plateau possesses a significant reservoir of halogenated organic compounds (HOCs). Owing to the permafrost’s properties as hydrological barriers,^6^ these compounds can be effectively stored for decades. However, permafrost thawing due to climate warming is expected to alter this scenario. Based on observations of dramatic climate warming over the past few decades, the Tibetan Plateau has suffered severe permafrost degradation,^1^ leading to the loss of hydrological barriers.^6^ Consequently, certain harmful substances present in permafrost might be released, thereby entering the aquatic environment. The Tibetan Plateau, often dubbed the Asian water tower, stands as one of the Earth’s most significant water distribution systems.^11−13^ Rivers that originate from the Tibetan Plateau provide water resource for the livelihoods of nearly two billion people and play an important role in local ecosystems.^14,15^ Therefore, when harmful compounds present in permafrost enter the aquatic environment, they might pose a substantial risk.

In the context of water quality, substances classified as persistent, mobile, and toxic (PMT) and very persistent and very mobile (vPvM) warrant particular attention.^16,17^ PMT/vPvM substances are difficult to be degraded and sorbed, and thus could persist for long time scales in the aquatic environments.^18,19^ Additionally, their mobility enables them to spread fast in aquatic systems and subsurface environments, and often through barriers used for water treatment.^18^ For these reasons the European Commission recently introduced PMT and vPvM substances as new hazard categories within the framework of the Classification, Labeling, and Packaging (CLP) regulation.^20,21^ In the interest of water safety, it is of importance to prioritize such chemicals present in the permafrost on the Tibetan Plateau in order to evaluate the impact introduced by thawing permafrost on water quality.

To fill this gap, our research aimed to identify PMT/vPvM substances among the recently detected compounds stored in the permafrost of the Tibetan Plateau. This analysis was prompted as many HOCs are known to be persistent in the environment.^22−24^ The specific tasks are (i) to identify the PMT/vPvM substances in the permafrost by combining the standard screening approach (for substances with high-quality experimental data) and a machine learning model (for substances missing reliable experimental data) and (ii) to analyze and compare molecular features of PMT/vPvM substances of synthetic or natural/undefined origin in the permafrost.

## 2 Methods and Materials

### 2.1 Chemical List for Analysis

Detected substances in the permafrost of the Tibetan Plateau were compiled from a review of 21studies found via a search in the ISI Web of Science (Text S1 and Tables S1 and S2).^10^ This list contains a total number of 542 organic compounds, including 287 synthetic compounds, 130 natural compounds, and 125 compounds with unknown sources. Monitoring data for soils of depth 0–150 cm were selected, as the identified studies mainly sampled from shallow active layer soils at the top of the permafrost, as shown in Figure S1.

### 2.2 PMT/vPvM Screening Approach Based on Experimental Data

A recent list^21^ of PMT/vPvM substances was consulted to ascertain which of the 542 target compounds had been previously evaluated (see Table S3). Second, for substances not evaluated, we conducted a sequential assessment of persistency, mobility, and toxicity according to the methodology of our previous study^25^ (see Table S3). Specially, the criteria for persistency (P) are derived from the EU CLP regulation,^21^ utilizing the half-life values of compounds in water. Briefly, the cutoff values for persistent (P) and very persistent (vP) categories are established at half-life times of 40 and 60 days in fresh water, respectively. Concerning mobility assessment, experimentally determined organic carbon–water distribution coefficient (log Koc) values (at pH 4–9) are utilized following the criteria proposed by the EU CLP regulation.^21^ These thresholds are categorized as follows: substances with a value <3.0 are classified as mobile (M), while those with a value <2.0 are deemed very mobile (vM). The toxicity criterion (T) is derived from EU CLP regulation^21^ for the assessment of PMT substances. These considerations include if a substance is carcinogenic, mutagenic, or toxic to reproduction, as well as toxic to aquatic organisms, exhibits specific target organ toxicity - repeated exposure (STOT RE), or has endocrine-disrupting properties. It should be noted that this approach should be considered a screening approach, as it does not follow the full weight of evidence protocols described in the CLP guideline.

### 2.3 PMT/vPvM Screening Approach Based on Machine Learning

For compounds without available experimental data, PMT/vPvM properties were predicted using our developed machine learning model.^26^ The outcome is presented in Table S4. Notably, the model was retrained using the data set classified according to the updated PMT/vPvM selection criteria recently proposed by the EU CLP regulation.^21^ The machine learning model could predict the PMT/vPvM property of target compounds based on the structures of compounds. Specifically, 288 molecular descriptors were selected to describe the structural information on compounds. All the molecular descriptors were calculated using the software alvaDesc (v2.0; Kode Chemoinformatic, Pisa, Italy) based on SMILES (Simplified Molecular Input Line Entry System). The SMILES codes for the chemicals can be sourced from PubChem (https://pubchem.ncbi.nlm.nih.gov/). The model outcomes were interpreted by utilizing the SHapley Additive exPlanations (SHAP) method,^27^ a model interpretation approach based on game theory. Additionally, the Molecular ACCess Systems keys (MACCS) molecular fingerprints (MFs) of identified PMT/vPvM substances were calculated based on the software alvaDesc (see Text S1).

### 2.4 Applicability Domain

With such models it is imperative to ensure target compounds are within the applicability domain (AD) prior to PMT/vPvM predictions by applying our machine learning model.^26^ AD was ascertained by assessing the similarity between the target compound and those present in the training data set, utilizing Euclidean distance as a metric. Specifically, a lower Euclidean distance indicates greater similarity. The Euclidean distances between all the target compounds and the compounds in the training set were lower than the threshold value of AD (see Table S4), indicating that all the compounds were within AD.

## 3 Results and Discussion

### 3.1 PMT/vPvM Substances in the Active Soil of Permafrost

Identifying PMT/vPvM substances among the identified compounds in the active soil of permafrost followed a three-step procedure, as elaborated in the Methods and Materials section. First, known PMT/vPvM substances among the compounds were identified.^21^ Second, a standard assessment protocol^21,25^ was applied for the remaining compounds, to see if they fulfilled the criteria for being persistent (P), very persistent (vP), mobile (M), very mobile (vM), and/or toxic (T). Finally, the suspected PMT/vPvM substances lacking high-quality experimental data were screened by our recently developed machine learning model to fill data gaps.^26^

**Figure 1:**
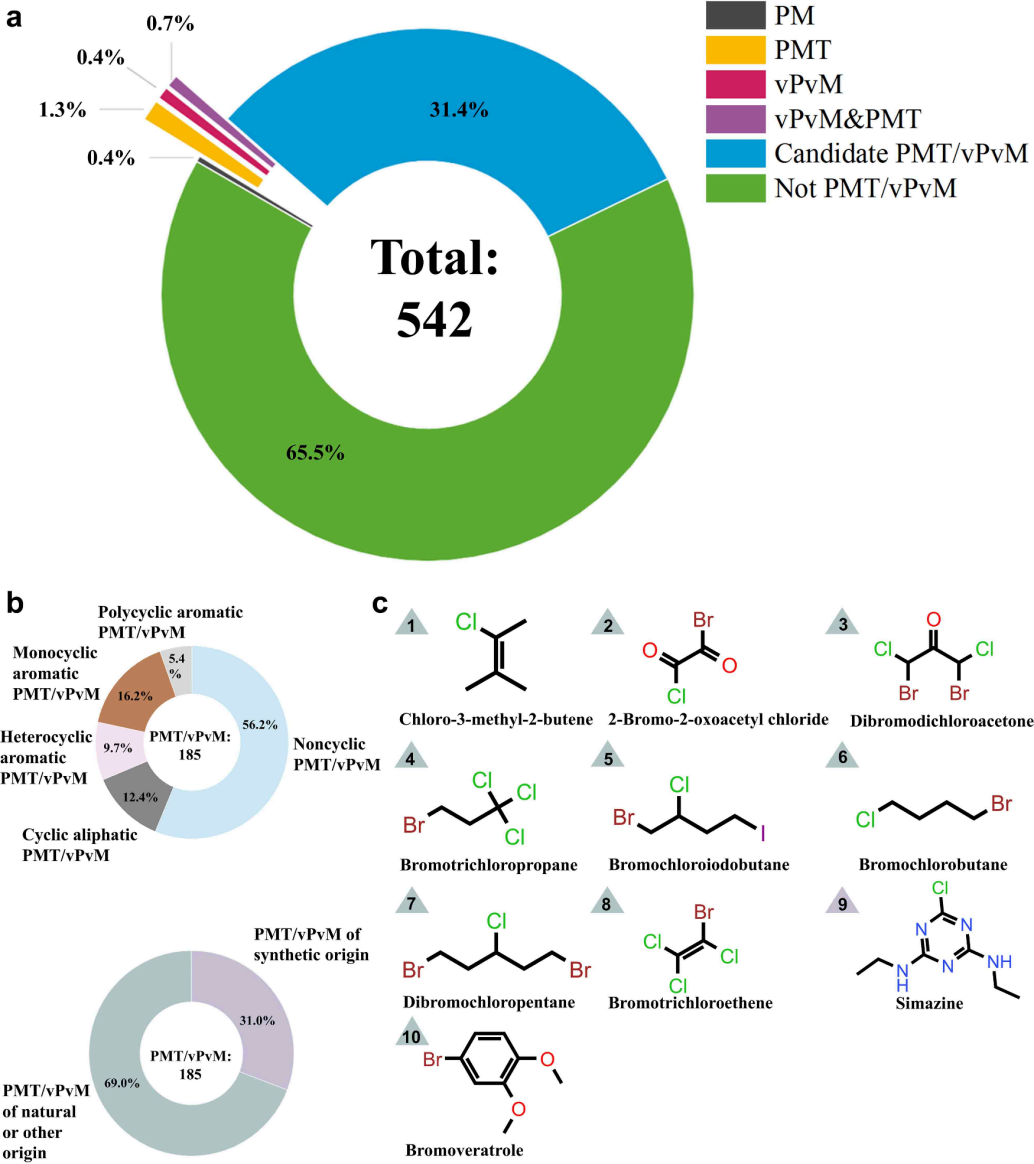
PMT/vPvM assessment of 542 compounds detected in the active soil of permafrost. (a) Classification of the target compounds criteria. (b) Distribution of PMT/vPvM substances from different sources as well as of different molecular features. (c) Molecular structures of the PMT/vPvM substances with the highest concentrations (top 10, with ranking shown).

Among the 542 identified compounds detected in the surface permafrost, 185 of them were previously assessed (see Figure 1 and Tables S3 and S4), including PMT substances (count 7), vPvM substances (count 4), PMT&vPvM substances (count 4), and candidate PMT/vPvM substances (count 170). Hereby, the candidate PMT/vPvM substances represent the predicted PMT/vPvM substances based on our machine learning model.^26^Figure S2 illustrates the molecular structures of PMT, vPvM, and PMT&vPvM substances. Notably, all the compounds are exclusively noncyclic, with the majority of them being poly/perfluoroalkyl substances (PFASs) and organophosphate esters. Of the entire set of 185 PMT/vPvM substances, noncyclic compounds also constituted a majority (56.2%) (Figure 1b). Approximately 69% of the PMT/vPvMs are considered to be of natural or other origin (Figure 1b), indicating that natural PMT/vPvM substances also require an emerging amount of attention. The concentration values of these 185 PMT/vPvM substances as reported could range over 1–670075 ng/g dry weight (dw). It is noteworthy to mention that Zhu et al.^10^ extracted HOCs from nonextractable residues (NERs) apart from the extractable fraction (EF) compared with other previous studies (see Text S1). As shown in Figure S3, concentrations of PMT/vPvM substances in NER (average 195133 ng/g, dw, range 6030–669943 ng/g, dw) are much higher than those found in the EF (average 154 ng/g, dw, range 0–1555 ng/g, dw). These sampling sites covered a large range of latitudes, and the concentrations of PMT/vPvM substances in the surface permafrost rose slightly from the north to the south (see Table S5). There were 10 PMT/vPvM substances that are among the most abundant HOCs detected in the active soil of permafrost, which were all from Zhu et al.^10^ (Figure 1c). These results suggest that several PMT/vPvM substances occur in the active soil of permafrost, including the most abundant compounds, potentially presenting a risk on the regional water quality due to their substantial chemical flux associated with permafrost thawing.

### 3.2 Molecular Features of PMT/vPvM Substances in the Active Soil of Permafrost

In order to explore the relationship between molecular structures and PMT/vPvM property, the Molecular ACCess Systems keys^28^ (MACCS) molecular fingerprints (MFs) of these identified PMT/vPvM substances were calculated (Table S6). Table 1 showed the top-10 molecular fingerprints with the highest number of occurrences in PMT/vPvM substances. Specifically, the most prevalent substructures observed in PMT/vPvM substances from the active soil of permafrost contained halogens (#134, 107, 103, 87, 46), rings (#165, 163), methyl groups (#160), branches (#107, 112), oxygen atom (#164, 157, 159, 152, 154, 139), and aromatic structures (#162) (see Table 1 and Table S6). According to a previous study,^29^ substances dominated by halogens, aromatic rings, alkyl-cyclical structures, and branched methyl groups tend to be associated with slow biodegradation rates, which increases the likelihood of such substances being more persistent in the environment. Additionally, polar functional groups (e.g., the number of oxygens or heteroatoms with diverse electronegativity)^30,31^ and the degree of branching^30^ can enhance the hydrophilicity of compounds, thus improving their mobility in aquatic systems. Our recent research^32^ indicated that the aromatic moiety may exert a positive causal effect on both the persistence and mobility of a substance (for smaller molecules).

**Table 1:** Top-10 Molecular Fingerprints of Compounds with the Highest Number of Occurrences in Persistent, Mobile, and Toxic (PMT) or Very Persistent and Very Mobile (vPvM) Substances

Identified PMT/vPvM				PMT/vPvM of synthetic origin				PMT/vPvM of natural or other origin			
Feature number^a^	Feature name	Description	Proportion^b^	Feature number^a^	Feature name	Description	Proportion^b^	Feature number^a^	Feature name	Description	Proportion^b^
134	X	Halogens	98%	134	X	Halogens	96%	134	X	Halogens	99%
107	XA(A)A	Halogen atom connected to one atom which is connected to two atoms	93%	107	XA(A)A	Halogen atom connected to one atom which is connected to two atoms	88%	107	XA(A)A	Halogen atom connected to one atom which is connected to two atoms	95%
103	Cl	Chlorine atom	84%	164	O	Oxygen atom	70%	103	Cl	Chlorine atom	93%
164	O	Oxygen atom	57%	112	AA(A)(A)A	One central atom connected to four atoms	68%	46	Br	Bromine atoms	52%
165	Ring	Any cyclic structure	44%	103	Cl	Chlorine atom	63%	164	O	Oxygen atom	52%
87	X!A$A	Halogen atom not bonded to an atom participating in a ring bond	41%	106	QA(Q)Q	One atom connected to three nonmethine groups	53%	165	Ring	Any cyclic structure	46%
46	Br	Bromine atoms	41%	159	O>1	More than one oxygen atom	51%	87	X!A$A	Halogen atom not bonded to an atom participating in a ring bond	44%
155	A!CH2!A	Methylene connected to other atoms with more than one chain bond	36%	157	C-O	Carbon–oxygen single bond	47%	162	Aromatic	Aromatic structure	39%
157	C-O	Carbon–oxygen single bond	36%	165	Ring	Any cyclic structure	39%	155	A!CH2!A	Methylene connected to other atoms with more than one chain bond	38%
153	QCH2A	Heteroatom connected to a methylene group that is bonded to another atom	35%	153	QCH2A	Heteroatom connected to a methylene group that is bonded to another atom	37%	150	A!A$A!A	Atom bridging two nonaromatic ring systems	34%

a The numbers are their original orders in the MACCS naming system.^28,33^

b This percentage represents the proportion of compounds that contain this molecular fingerprint to the total number of identified compounds. A: any valid periodic table element symbol; Q: heteroatoms, any non-C or non-H atom; X: halogens; other letters are referring to periodic table elements or organic molecular fragments (containing C and H); =: double bond; $: ring bond; !: chain or nonring bond; %: aromatic bond.

**Figure 2:**
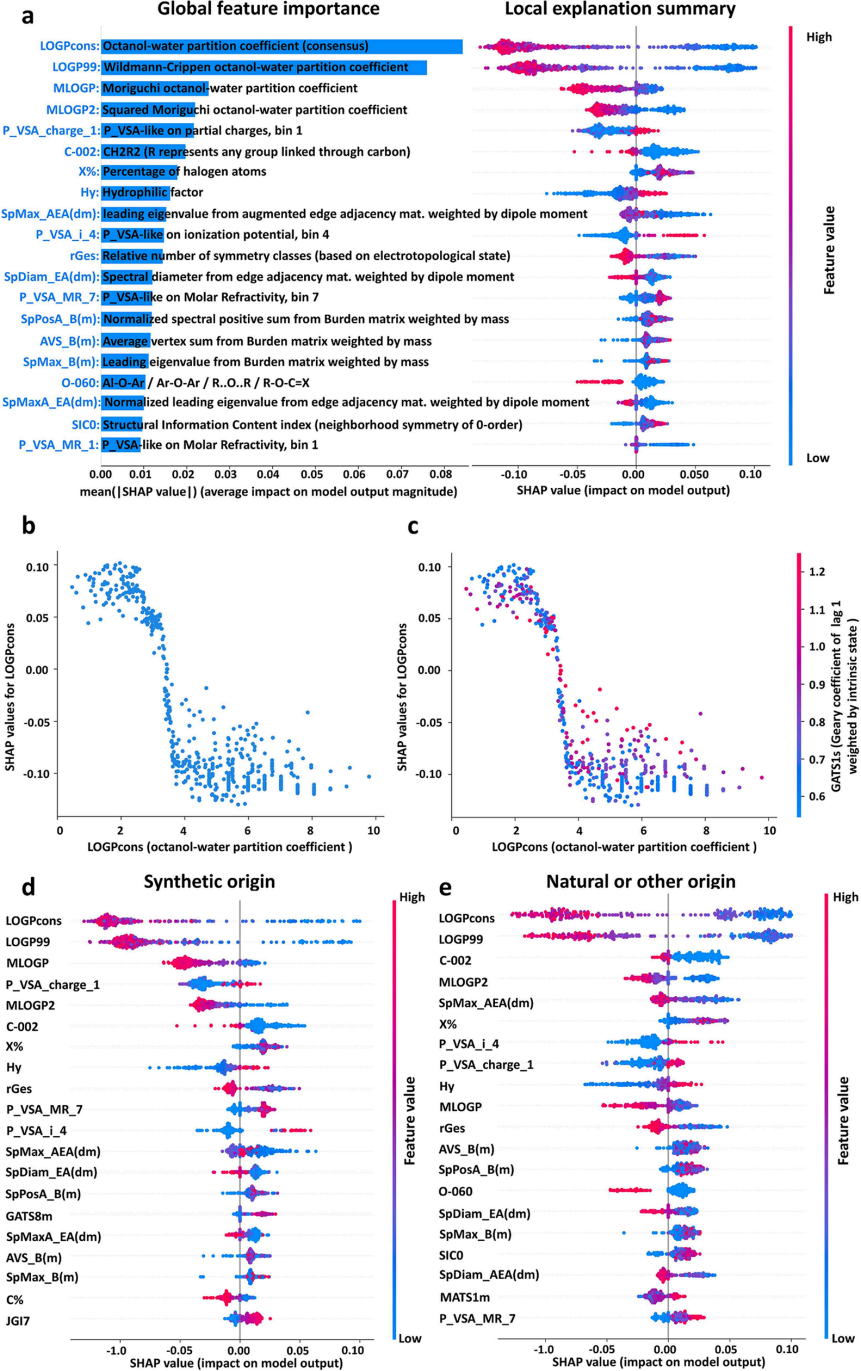
(a) Left: bar chart of the average impact on model output magnitude, which denotes feature importance values for our model. Right: beeswarm plot of our model on 542 compounds detected in the active soil of permafrost. (b) SHAP dependence plot of LOGPcons versus its SHAP value. (c) Plot of the SHAP interaction value of LOGPcons with AVS_B(m). The *x*-axis is the value of the feature, and the *y*-axis is the SHAP value for that feature. The color corresponds to a second feature that may have an interaction effect with the feature. (d) Beeswarm plot of our model on synthetic-like compounds detected in the active soil of permafrost. (e) Beeswarm plot of our model on natural and unknown origin compounds detected in the active soil of permafrost. The detailed information of these molecular descriptors is summarized in Table S8.

The Shapley additive explanations (SHAP) method^27^ was applied to further explore common features of the identified PMT/vPvM substances. As demonstrated in Figure 2a, the most important molecular descriptors were sequentially presented from top to bottom based on their influence on model predictions. The detailed information on these molecular descriptors was summarized in Text S3 and Table S8. Specifically, the molecular descriptor LOGPcons was presented uppermost, signifying that this descriptor is contributing most to predictions of PMT/vPvM properties in the model. LOGPcons represents the octanol–water partition coefficient (log P). As shown in Figure 2b, LOGPcons exhibit a negative correlation with their SHAP values, implying that decreasing LOGPcons increases the likelihood of PMT/vPvM properties. For the mobility consideration, this is expected as a lower LOGPcons indicates increased hydrophilicity of chemicals. It should also be considered that, because HOCs are in general considered persistent,^22−24^ this would result in a mobility related parameter being an important indicator of PMT/vPvM properties. However, even among substances with similar LOGPcons, the SHAP values of the different compounds vary, indicating an interrelationship with other molecular descriptors. Specifically, LOGPcons interacted most with the molecular descriptor GATS 1s (Geary autocorrelation of lag 1 weighted by I-state), which related to the molecular size.^34^Figure 2c presents that GATS 1s increases along with decreasing SHAP values of LOGPcons, indicating that a higher molecular size at a given LOGPcons would decrease the likelihood of a substance with PMT/vPvM properties.

### 3.3 Characteristics of PMT/vPvM Substances of Different Origin in the Active Soil of Permafrost

Zhu et al.^10^ characterized individual HOCs in the Tibetan permafrost as coming from either synthetic origin, natural sources, and undefined sources, with the differentiation based on the plausibility that a substance could be formed from a synthetic or natural process or not. An example of how an HOC can be formed from natural processes is when industrial (or natural) emissions of reactive chlorine^35^ interact with volatile organic carbonaceous substances, that ultimately undergo atmospheric deposition.^36^ Many of the HOCs in Tibetan permafrost, however, predate industrial times and would have been formed by naturally occurring emissions of reactive chlorine. To characterize differences in the PMT/vPvM substances that fall into these origin categories, the relevant molecular fingerprints were calculated and compared (Table 1 and Table S6). For the sake of simplicity, the substances from natural and undefined sources were grouped. Among the top 20 molecular fingerprints, there are 7 molecular fingerprints that are inconsistent between PMT/vPvM substances of synthetic and natural/undefined origin (see Table S7). Generally, PMT/vPvM substances of synthetic origin showed a higher proportion of fluorine (#42), branched structures (#112), and heteroatoms (#106, 124, 148, 102). Due to the extremely strong electronegativity of the fluorine element, elemental fluorine readily forms fluoride ions which do not readily react to form organic compounds in natural processes.^37^ Therefore, the content of natural fluorinated compounds is very low.^37^ On the other hand, the synthetic PFAS detected commonly contain complex branched chains and multiple substituent groups, which highly coincide with the patterns of AA(A)(A)A (one central atom connected to four atoms) and QA(Q)Q (one atom connected to three nonmethane groups) in the MACCS fingerprint. The seven unique molecular fingerprints that only appeared in the top-20 molecular fingerprints of natural/undefined origin of PMT/vPvM substances were bromine atoms (no. 46), aromatic structure (no. 162), carbon–oxygen bonds (no. 152, no. 154), methyl groups (no. 160), and rings (no. 144, no. 143), respectively (see Table 1 and Table S7). As shown in Table 1, the proportions of occurrence for chloride and bromide in natural PMT/vPvM substances are higher than those in synthetic PMT/vPvM substances. PMT/vPvM substances of natural/undefined origin also exhibit a higher occurrence of carbon–oxygen bonds, perhaps reflective of the abundance of carbonyls generated from haze pollution.^38^

Figure 2d,e demonstrates the top-20 significant molecular descriptors for PMT/vPvM HOCs of synthetic and natural/identified origin, respectively. The detailed information of these molecular descriptors is summarized in Table S8. Synthetic origin PMT/vPvM substances exhibit four distinctive molecular features that are different from the natural/undefined ones: GATS8m (Geary autocorrelation of lag 8 weighted by mass) and SpMaxA_EA(dm) (normalized leading eigenvalue from edge adjacency mat. weighted by dipole moment), C% (percentage of C atoms), and JGI7 (mean topological charge index of order 7). Specifically, GATS8m is a common 2D autocorrelation molecular descriptor in cheminformatics, used to reflect the distribution of atomic mass and numbers in molecular topological structures.^39^ As shown in Figure 2d, GATS8m was positively correlated to PMT/vPvM properties. This finding is consistent with the previous study that GATS8m positively affected the toxicity of organophosphate esters.^39^ SpMaxA_EA(dm) is a molecular descriptor related to the dipole moment of a molecule. As shown in Figure 2d, SpMaxA_EA(dm) negatively contributes to PMT/vPvM properties of compounds of synthetic origin. According to a previous study,^40^ SpMaxA_EA(dm) is positively correlated with degradation rates of pharmaceuticals and pesticides, thereby reducing the persistence of these compounds in the environment. This agrees well with our model interpretation results. Additionally, C% represents the percentage of C atoms and JGI7 is a molecular descriptor that considers the charge distributions of these molecules.^41^ These results indicated that PMT/vPvM properties of synthetic compounds are more closely associated with decreased dipole moment, decreased percentage of C atoms, increased charge distribution heterogeneity, and increased distribution of atomic mass heterogeneity compared with natural compounds.

There were four molecular features that only appeared in natural/undefined origin compounds: O-060 (Al-O-Ar/Ar-O-Ar/R..O..R/R-O-C=X, R represents any group linked through carbon; X represents any heteroatom; Al and Ar represent aliphatic and aromatic groups, respectively; = represents a double bond), SIC0 (Structural Information Content index (neighborhood symmetry of 0-order)), SpDiam_AEA (dm) (spectral diameter from augmented edge adjacency mat. weighted by dipole moment), and MATS1m (Moran autocorrelation of lag 1 weighted by mass). Specifically, the values of the O-060 and SIC0 for compounds of natural or unknown origin were higher in comparison to those of synthetic origin. Conversely, the SpDiam_AEA (dm) and MATS1m values were notably lower than those observed in compounds of synthetic origin. As an atom-centered fragment, O-60 represented specific molecular structures containing oxygen atoms, such as aromatic ether (see Text S3). SIC0 is a normalized form of information entropy, which quantifies the diversity of the type distribution of atoms in a molecule (Text S3). SpDiam_AEA(dm) is a topological descriptor derived from the edge adjacency matrix, which is sensitive to charge separation in a molecule.^42^ MATS1m is a molecular descriptor for quantifying the similarity in the mass of adjacent atoms. As shown in Figure 2e, molecules with higher MATS1m values are more likely to be PMT/vPvM substances. This is in line with the previous study that MATS1m has a significant positive effect on the toxicity.^43^ To summarize, the PMT/vPvM properties of natural compounds are more sensitive to increasing molecular complexity, decreasing charge separation heterogeneity, and increasing adjacent atomic mass similarity.

## 4 Environmental Implications

This study identified compounds that are PMT/vPvM substances or candidates thereof in the active layer of degrading Tibetan permafrost. An important consideration going forward is how much of these substances would be released in a warming climate. The permafrost thaw induced by seasonal temperature changes and/or global warming could open new hydrological pathways^44,45^ and change hydrologic connectivity,^6^ resulting in release and dispersal of PMT/vPvM substances. Zhu et al.^10^ differentiated between easily extractible fractions that could be released rapidly and NERs that would be released more slowly over time. In particular, Zhu et al.^10^ noted that the 20 μg/g of aliphatic and monocyclic aromatic NERs-HOCs could be remobilized when the temperature increased from −4 to −2 °C. Future work should try to simulate such emissions of PMT/vPvM substances from this Asian Water Tower to the downstream water reservoirs, to better anticipate the impacts of permafrost degradation.^46^ It is hoped that this work can provide valuable information to guide prioritization strategies for monitoring impacts on the fluxes of HOCs and regional water quality, resulting from the melting of the active layer of permafrost, by focusing on PMT/vPvM substances. Future work on this topic could also combine machine learning and nontarget analysis approaches in order to identify the unknown PMT/vPvM substances in the thawing permafrost.

## Supporting Information Available

The detailed description of data collection (Text S1); the methods to calculate MACCS fingerprints and top-20 molecular descriptors (Text S2); distribution of sampling depth in the Tibetan Plateau (Text S3); the molecular structures of PMT substances (Figure S1); vPvM substances and PMT&vPvM substances (Figure S2); the concentration distribution of PMT/vPvM substances in the surface permafrost soils (Figure S3); the parameters of the MLOGP model (Table S9); van der Waals radii used for VSA calculations (Table S10); reference bond lengths used for VSA calculation (Table S11) (PDF)
Literature consulted regarding the compilation of detected compounds in soil of the Tibetan Plateau (Table S1); compounds detected in the active soil layer of the Tibetan Plateau (Table S2); list of compounds with high-quailty experimental data for the PMT/vPvM hazard class evaluation (Table S3); model predictions of compounds without high-quality experimental data for the PMT/vPvM hazard class evaluation (Table S4); the concentration and distribution of PMT/vPvM substances on the active soil layer of Tibetan permafrost (Table S5); the MACCS molecular fingerprints of the PMT/vPvM substances in permafrost (Table S6); the top-20 MFs with the highest number of occurrences in PMT/vPvM substances (Table S7); and the detailed information of the top-20 important molecular descriptors (Table S8) (XLSX)
