# Supplementary material for: The Active Soil Layer of Thawing Permafrost Is an Emergent Source for Organic Substances of Concern to Water Resources
Source: Environ Sci Technol Lett. 2025 Apr 21;12(5):558–66. doi: 10.1021/acs.estlett.5c00275 (PMC12080254; doi:10.1021/acs.estlett.5c00275)
Supplement: Supplementary file 1 — ez5c00275_si_001.pdf [file ez5c00275_si_001.pdf]

Supporting Information for

The active soil layer of thawing permafrost is an emergent source for organic substances of concern to water resources

Min Han[a,b,c], Biao Jin[a,b,c*], Hans Peter H. Arp[d,e*]

[a]State Key Laboratory of Advanced Environmental Technology, Guangzhou Institute of Geochemistry, Chinese Academy of Sciences, Guangzhou, 510640, China

[b]University of Chinese Academy of Sciences, Beijing 10069, China

[c]Guangdong Provincial Key Laboratory of Environmental Protection and Resources Utilization, Guangzhou 510640, China

[d]Norwegian Geotechnical Institute (NGI), P.O. Box 3930 Ullevaal Stadion, N-0806 Oslo, Norway

[e]Norwegian University of Science and Technology (NTNU), NO-7491 Trondheim, Norway

*__Corresponding author:__ *Biao Jin, jinbiao@gig.ac.cn; Hans Peter H. Arp, hans.p.arp@ntnu.no*

This document includes:

18 pages

3 Figures (Figures S1 – S3)

3 Tables (Tables S9 – S11, Tables S1-S8 can be found on the accompanying xlsx file)

Text S1. Data collection

We conducted a comprehensive search of the Web of Science for the literature published from January 1991 through February 2025, using keywords related to soils on the Tibetan Plateau and organic compounds (Table S1). Organic compounds detected and identified in soils on the Tibetan Plateau were extracted from this literature[1-21] (Table S2). Furthermore, the concentrations of compounds in soils, sampling depth, and latitude and longitude coordinates for each site, extracted from published articles. Note that Zhu et al.[1] extracted HOC through accelerated solvent extraction, employing a sequence of solvents, namely acetone, acetone/hexane, and n-hexane. This approach appears to be robust in enhancing the solubility of molecules. However, it should be noted that chemical extraction processes, including those involving BBr3 treatment, carry the risk of generating chemical artifacts.

.

Text S2. The calculation of MACCS fingerprints

In order to explore the relationship between molecular structures and PMT/vPvM property, the Molecular ACCess Systems keys[22] (MACCS) molecular fingerprints (MFs) of these identified PMT/vPvM substances were calculated using the software, alvaDesc (v2.0; Kode Chemoinformatic, Pisa, Italy) based on SMILES (Simplified Molecular Input Line Entry System). The MACCS 166 fingerprint is a fixed-size boolean vector reflecting the presence/absence of a set of 166 well defined molecular features. It has been widely used for drug discovery and virtual screening. The alvaDesc software is one of the most recent tools for the calculation of molecular descriptors and fingerprints.

Text S3. The methods to calculate top-20 molecular descriptors

All the molecular descriptors were also calculated using the software, alvaDesc (v2.0; Kode Chemoinformatic, Pisa, Italy) based on SMILES (Simplified Molecular Input Line Entry System). In order to better understand the meaning of molecular descriptors, the methods to calculate the top-20 MDs were presented as following:

(1) *LOGPcons* represents the octanol-water partition coefficients based on a LogP consensus model. Specifically, LOGPcons is the mean value of three octanol-water partition coefficients, including Moriguchi octanol-water partition coefficient (*MLOGP*), the Ghose-Crippen-Viswanadhan octanol-water partition coefficient (*ALOGP*)[23] and the Wildman-Crippen octanol-water partition coefficient (*LOGP99*)[24].

$$LOGPcons = \frac{MLOGP + ALOGP + LOGP99}{3} \quad \text{(S1)}$$

MLOGP model is defined as:

$$MLOGP = -1.041 + 1.244 \cdot CX^{0.6} - 1.017 \cdot NO^{0.9} + 0.406 \cdot PRX - 0.145 \cdot UB^{0.8} + 0.511 \cdot HB + 0.268 \cdot POL - 2.215 \cdot AMP + 0.912 \cdot ALK - 0.392 \cdot RNG - 3.684 \cdot QN + 0.474 \cdot NO2 + 1.582 \cdot NCS + 0.773 \cdot BLM \quad \text{(S2)}$$

The description of the 13 structural parameters in eq. S2 was summarized in Table S9.

ALOGP and *LOGP99* models are defined as:

$$LOGP = \sum_i n_i \cdot h_i \quad \text{(S3)}$$

where *ni* is the number of atoms of type *i* and hi is the corresponding hydrophobicity contribution.

(2) *LOGP99* is calculated as the sum of the contributions of each of the atoms in the molecules. The definition of *LOGP99*[24] was presented in eq. S3.

(3) *MLOGP* is a regression model based on 13 structural parameters. The regression

coefficients were evaluated by a training set of 1,230 organic molecules resulting in a R2 equal to 0.906. Training set includes aliphatic, aromatic, and heterocyclic compounds, containing the following atom types: C, H, N, O, S, P, F, Cl, Br, I. The definition of *MLOGP* is presented in eq. S2.

(4) *MLOGP2* represents the square value of *MLOGP*:

$$MLOGP2 = MLOGP^2 \tag{S4}$$

(5) P_VSA_charge_1(P_VSA-like on partial charges, bin 1) belongs to P_VSA-like descriptors, which are based on the sum of atomic contributions due to the van der Waals surface area[25].

$$P\_VSA\_charge\_1 = \sum_{i=1}^{nAT} VSA_i \cdot \delta(charge_i \epsilon(-\infty, -0.3)) \tag{S5}$$

where：

- nAT is the number of atoms
- δ: is a Kronecker delta function equal to one for atoms with property value in the specified range, and zero otherwise
- $charge_i$ is the partial charge of the atom i
- $VSAi$ is the van der Waals surface area of the i-th atom：

$$VSA_{\mathrm{i}} = 4 \cdot \pi \cdot R_i^2 - \pi \cdot R_i \cdot \sum_{j=1}^{nAT} a_{ij} \cdot \left(\frac{R_j^2 - (R_i - d_{ij})^2}{d_{ij}}\right) \tag{S6}$$

where Ri is the atomic van der Waals radius of the atom i as defined in Table S10, nAT is the number of atoms, a_{ij} are the elements of the adjacency matrix and d_{ij} is calculated as:

$$d_{ij} = \min\{\max\{|R_i - R_j|, b_{ij}\}, R_i + R_j\} \tag{S7}$$

where b_{ij} is the ideal bond length between atom i and j and it is defined as:

$$b_{ij} = r_{ij} - c_{ij} \tag{S8}$$

where r_{ij} is the reference bond length as defined in Table S11 and c_{ij} is a correction term related to the bond multiplicity: 0 for single bond, 0.1 for aromatic, 0.2 for double and 0.3 for triple bonds.

(6) C-002 belongs to atom-centred fragments, which are simple molecular descriptors defined as the number of specific atom types in a molecule. Specifically, C-002 represents number of the molecular structure “CH2R2” in a molecule, where R

represents any group linked through carbon.

(7) *X%* is percentage of halogen atoms:

$$X\% = \frac{nX}{nAT} \quad \text{(S9)}$$

where *nX* is the number of halogen atoms and *nAT* is the number of atoms.

(8) Hy[26] is the hydrophilic factor which is a hydrophilicity descriptor defined as:

$$Hy = \frac{(1+N_{Hy})\cdot log_2(1+N_{Hy})+nC\cdot\left(\frac{1}{nSK}\cdot log_2\frac{1}{nSK}\right)+\sqrt{\frac{N_{Hy}}{nSK^2}}}{\log_2(1+nSK)} \quad \text{(S10)}$$

where N_{Hy} is the number of hydrophilic groups (-OH, -SH, -NH), *nC* is the number of carbon atoms and *nSK* is the number of non-hydrogen atoms.

(9) *SpMax_AEA(dm)*, pertains to edge adjacency indices; the group of topological molecular descriptors derived from the edge adjacency matrix.

$$SpMax\_AEA(dm) = \max\{\lambda_i\} \quad \text{(S11)}$$

where λ is the eigenvalue of edge adjacency matrix (AEA). The edge adjacency matrix (AEA) is obtained from the edge adjacency matrix by replacing the zero diagonal elements of the edge adjacency matrix with specific bond properties.

$$[AEA(dm)]_{ij} = \begin{cases} 1, & if\ i,j\ are\ adjacent\ bonds \\ dm_i, & if\ i=j \\ 0, & otherwise \end{cases} \quad \text{(S12)}$$

where dm represents dipole moments.

(10) *P_VSA_i_4* (P_VSA-like on ionization potential, bin 4) belongs to P_VSA-like descriptors, which are based on the sum of atomic contributions due to the van der Waals surface area[25].

$$P\_VSA\_i\_4 = \sum_{j=1}^{nAT} VSA_j \cdot \delta(i_j \epsilon [1.25, +\infty)) \quad \text{(S13)}$$

- *nAT* is the number of atoms
- δ: is a Kronecker delta function equal to one for atoms with property value in the specified range, and zero otherwise
- i_j is the ionization potential of the atom *j*
- VSA_j is the van der Waals surface area of the j-th atom

(11) *rGes* is the relative number of classes on electrotopological state.

(12) *SpDiam_EA(dm)* is defined as:

$$\text{SpDiam\_EA(dm)} = \max\{\lambda_i\} - \min\{\lambda_i\} \quad \text{(S14)}$$

where λ is the eigenvalue of edge adjacency matrix (EA). The edge adjacency matrix (EA) is a square symmetric matrix of dimension nBO. The entries of the matrix equal one if the considered bonds are adjacent and zero otherwise.

(13) *P_VSA_MR_7* is defined as:

$$P\_VSA\_MR\_7 = \sum_{i=1}^{nAT} VSA_i \cdot \delta(MR_i \epsilon [4{,}6)) \quad \text{(S15)}$$

- *nAT* is the number of atoms
- δ: is a Kronecker delta function equal to one for atoms with property value in the specified range, and zero otherwise
- MR_i is the ionization potential of the atom *i*
- *VSAi* is the van der Waals surface area of the i-th atom

(14) *SpPosA_B(m)* represents normalized spectral positive sum from Burden matrix weighted by mass and is defined as follow:

$$SpPosA\_B(m) = \frac{1}{n} \cdot \sum_{i=1}^{n^+} (\lambda_i^+) \quad \text{(S16)}$$

where n is the number of non-H atoms and λ^+ is a positive eigenvalue of Burden matrix (*B(m)*). *B(m)* is a square symmetric matrix defined as follow:

$$[B(m)]_{ij} = \begin{cases} \frac{m_i}{m_C}, & if\ i = j \\ \sqrt{\pi_b} + 0.1, & if\ i\ and\ j\ are\ connected\ and\ one\ is\ a\ terminal\ atom \\ \sqrt{\pi_b}, & if\ \ i\ and\ j\ are\ connected \\ 0.001, & if\ \ i\ and\ j\ are\ not\ connected \end{cases}$$

(S17)

where *m* is the atomic mass and π_b is the bond order

(15) *AVS_B(m)* is the average vertex sum from Burden matrix weighted by mass:

$$AVS\_B(m) = \frac{1}{n} \cdot \sum_{i=1}^{n} \sum_{j=1}^{n} [B(m)]_{ij} \quad \text{(S18)}$$

where *n* is the number of non-H atoms and *B(m)* is a square symmetric matrix defined as eq. S18.

(16) *SpMax_B(m)* represents leading eigenvalue from Burden matrix weighted by mass:

$$SpMax\_B(m) = max_i\{\lambda_i\} \quad (S19)$$

where n is the number of non-H atoms and *B(m)* is a square symmetric matrix defined as eq. S18.

(17) O-060 belongs to atom-centred fragments, which are simple molecular descriptors defined as the number of specific atom types in a molecule. Specifically, O-060 represents number of the molecular structure "Al-O-Ar / Ar-O-Ar / R•••O •••R / R-O-C=X" in a molecule, where R represents any group linked through carbon; X represents any heteroatom (O, N, S, P, Se, and halogens); Al and Ar represent aliphatic and aromatic groups, respectively; = represents a double bond; : represents an aromatic bond as in benzene or delocalised bonds such as the N-O bond in a nitro group; •••, represents aromatic single bonds as the C-N bond in pyrrole.

(18) SpMaxA_EA(dm), pertains to edge adjacency indices; the group of topological molecular descriptors derived from the edge adjacency matrix

$$SpMaxA\_EA(dm) = \frac{SpMax\_EA(dm)}{nBO} \quad (S20)$$

where *nBO* is the number of bonds in an H-depleted molecular graph and (9) *SpMax_EA(dm)* is defined as:

$$SpMax\_EA(dm) = \max\{\lambda_i\} \quad (S21)$$

where λ is the eigenvalue of edge adjacency matrix (EA). The edge adjacency matrix (EA) is a square symmetric matrix of dimension nBO. The entries of the matrix equal one if the considered bonds are adjacent and zero otherwise.

$$[EA(dm)]_{ij} = \begin{cases} dm_j & if\ i,j\ are\ adjacent\ bonds \\ 0 & otherwise \end{cases} \quad (S22)$$

(19) *SIC0* is the structural information content index (neighborhood symmetry of 0-order), belonging to structural information content descriptor.

$$SIC0 = \frac{IC0}{\log_2 nAT} \quad (S23)$$

where *nAT* is the number of atoms and *IC0* is the neighborhood information Content defined as follow:

$$IC0 = -\sum_g \frac{n_g}{nAT} \cdot \log_2(\frac{n_g}{nAT}) \quad (S24)$$

where g runs over the equivalence classes, ng is the number of atoms belonging to the g-th equivalence class and nAT is the total number of atoms.

According to its definition, high SIC0 values (close to 1) indicate an evenly distributed atom types (such as C, N, O, S) and complex structures. Low SIC0 values (close to 0) represent single atom types (such as C and H only) and simple structures.

(20) *P_VSA_MR_1* is defined as:

$$P\_VSA\_MR\_1 = \sum_{i=1}^{nAT} VSA_i \cdot \delta(MR_i \epsilon(-\infty, 0.9)) \quad \text{(S25)}$$

- nAT is the number of atoms
- δ: is a Kronecker delta function equal to one for atoms with property value in the specified range, and zero otherwise
- MR_i is the ionization potential of the atom i
- $VSAi$ is the van der Waals surface area of the i-th atom

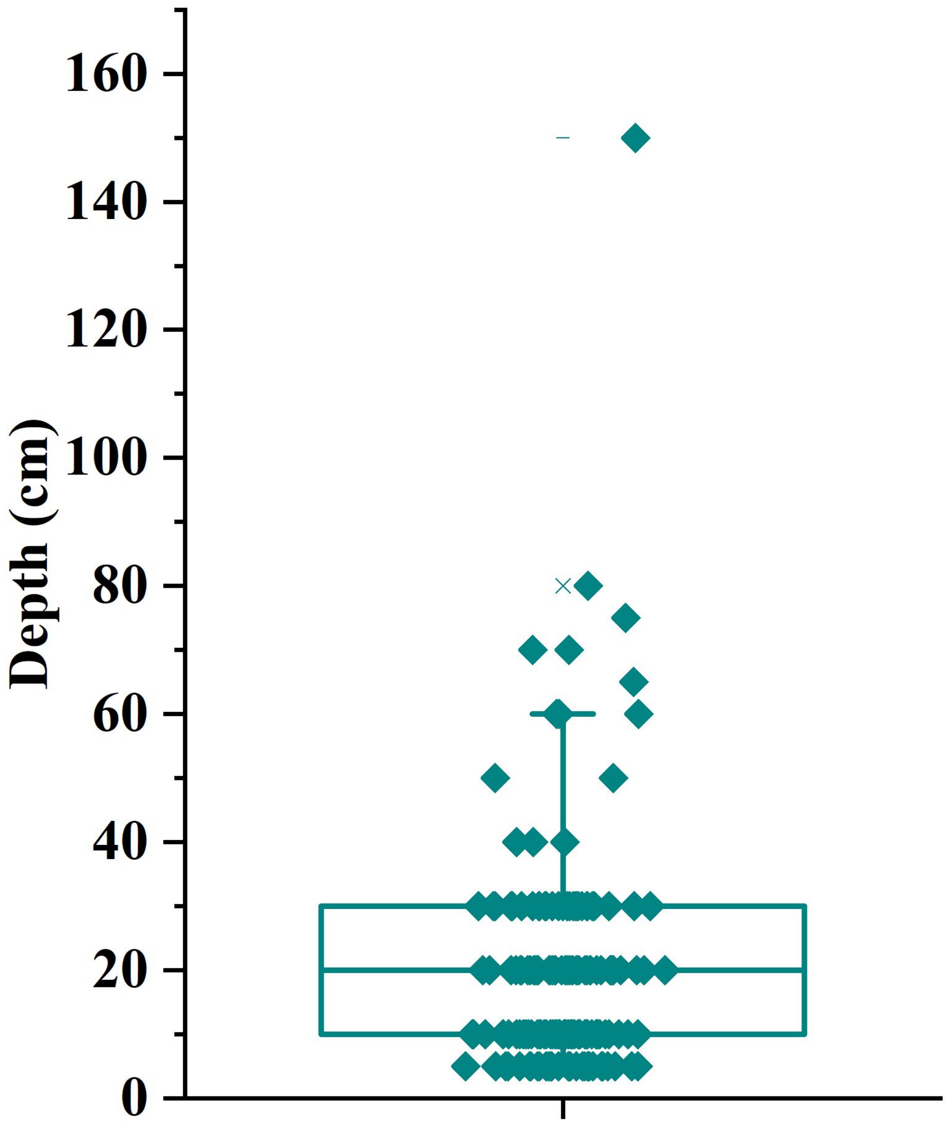

Fig. S1 Distribution of sampling depth in Tibetan Plateau.

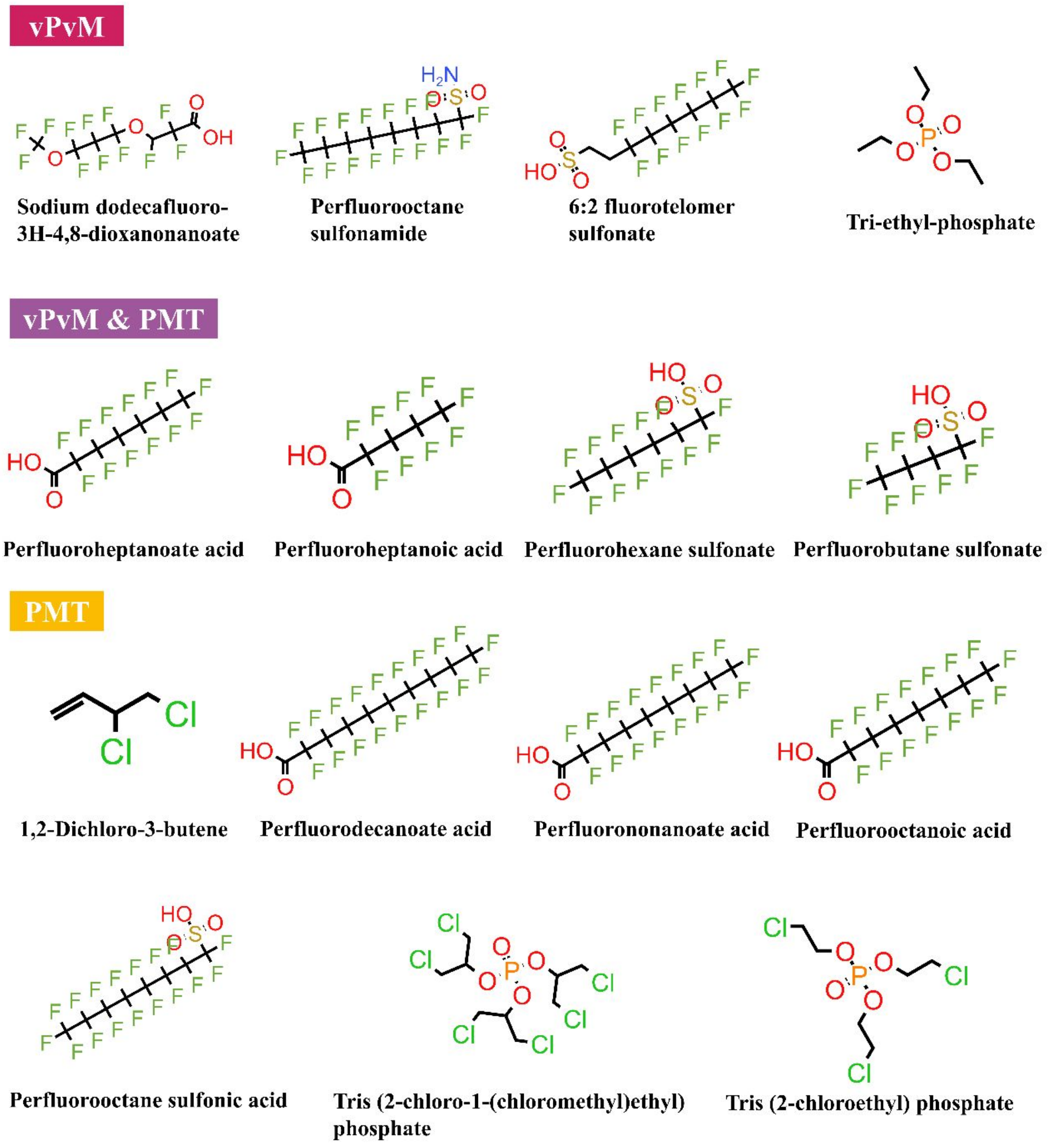

Fig. S2 The molecular structures of PMT substances (count 7), vPvM substances (count 4), and PMT&vPvM substances (count 4).

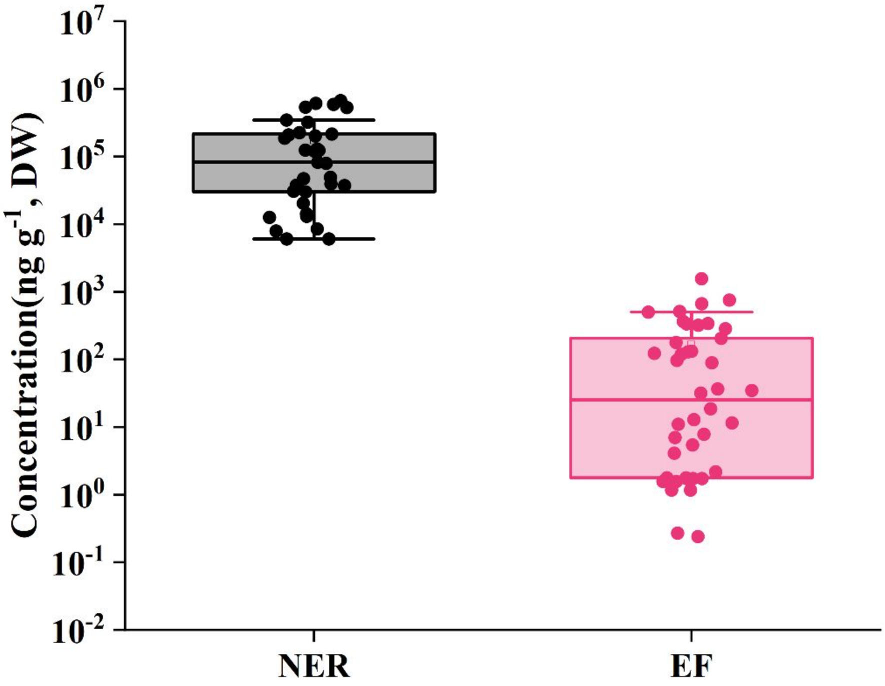

Fig. S3 Distribution of PMT/vPvM substances in the surface permafrost soils as EF and NER.

Table S9. The parameters of MLOGP model

Parameter	Description
CX	Summation of the number of carbon and halogen atoms weighted by: C: 1.0, F: 0.5, Cl: 1.0, Br: 1.5 and I: 2.0
NO	Total number of N and O atoms
PRX	Proximity effect of N/O; X-Y: 2.0, X-A-Y: 1.0 (X, Y: N/O, A: C, S or P) with a correction (-1) for carboxamide/sulfonamide
UB	Total number of unsaturated bonds except those in NO_2
HB	Dummy variable for the presence of intramolecular hydrogen bond as *ortho*-OH and -CO-R, -OH and -NH_2, -NH_2 and -COOH, or 8-OH/NH_2 in quinolines, 5 or 8-OH/NH_2 in quinoxalines, etc.
POL	Number of aromatic polar substituents (aromatic substituents excluding Ar-CX_2- and Ar-CX=C<, X: C or H). Upper limit = 4.
AMP	Amphoteric property; α-aminoacid: 1.0, aminobenzoic acid: 0.5, pyridinecarboxylic acid: 0.5.
ALK	Dummy variable for alkane, alkene, cycloalkane or cycloalkene (hydrocarbons with 0 or 1 double bond) or hydrocarbon chain with at least 7 carbon atoms
RNG	Dummy variable for the presence of ring structures except benzene and its condensed rings (aromatic, heteroaromatic, and hydrocarbon rings)
QN	Quaternary nitrogen >N^+<: 1.0; N oxide: 0.5
NO2	Number of nitro groups
NCS	Isothiocyanate (-N=C=S): 1.0; thiocyanate (-S-C≡N): 0.5
BLM	Dummy variable for the presence of ß-lactam

Table S10. Van der Waals radii used for VSA calculations (Radii are given in Angstroms)

Atom-type	R
H (–O)	0.8
H (–N, –P)	0.7
H (other)	1.485
C	1.950
N	1.950
O (oxide)	1.810
O (acid)	2.152
O (other)	1.779
F	1.496
P	2.287
S	2.185
Cl	2.044
Br	2.166
I	2.358

Table S11. Reference bond lengths used for VSA calculation (Lengths are given in Angstroms)

Bond-type	r	Bond-type	r
Br - Br	2.54	F - F	1.28
Br - C	1.97	F - H	0.87
Br - Cl	2.36	F - I	2.04
Br - F	1.85	F - N	1.41
Br - H	1.44	F - O	1.32
Br - I	2.65	F - P	1.5
Br - N	1.84	F - S	1.64
Br - O	1.58	H - I	1.63
Br - P	2.37	H - N	1.01
Br - S	2.21	H - O	0.97
C - C	1.54	H - P	1.41
C - Cl	1.8	H - S	1.31
C - F	1.35	I - I	2.92
C - H	1.06	I - N	2.26
C - I	2.12	I - O	2.14
C - N	1.47	I - P	2.49
C - O	1.43	I - S	2.69
C - P	1.85	N - N	1.45
C - S	1.81	N - O	1.46
Cl - Cl	2.31	N - P	1.6
Cl - F	1.63	N - S	1.76
Cl - H	1.22	O - O	1.47
Cl - I	2.56	O - P	1.57
Cl - N	1.74	O - S	1.57
Cl - O	1.41	P - P	2.26
Cl - P	2.01	P - S	2.07
Cl - S	2.07	S - S	2.05

Reference

(1) Zhu, X.; Yang, F.; Li, Z.; Fang, M.; Ma, S.; Zhang, T.; Li, C.; Guo, Q.; Wang, X.; Zhang, G.; et al. Substantial halogenated organic chemicals stored in permafrost soils on the Tibetan Plateau. *Nature Geoscience* **2023**, *16* (11), 989-996. DOI: 10.1038/s41561-023-01293-1.

(2) Wang, X.-p.; Sheng, J.-j.; Gong, P.; Xue, Y.-g.; Yao, T.-d.; Jones, K. C. Persistent organic pollutants in the Tibetan surface soil: Spatial distribution, air–soil exchange and implications for global cycling. *Environmental Pollution* **2012**, *170*, 145-151. DOI: https://doi.org/10.1016/j.envpol.2012.06.012.

(3) Ren, J.; Wang, X.; Gong, P.; Wang, C. Characterization of Tibetan Soil As a Source or Sink of Atmospheric Persistent Organic Pollutants: Seasonal Shift and Impact of Global Warming. *Environmental Science & Technology* **2019**, *53* (7), 3589-3598. DOI: 10.1021/acs.est.9b00698.

(4) Xing, X.-L.; Qi, S.-H.; Zhang, Y.; Yang, D.; Odhiambo, J. O. Organochlorine Pesticides (OCPs) in Soils Along the Eastern Slope of the Tibetan Plateau. *Pedosphere* **2010**, *20* (5), 607-615. DOI: https://doi.org/10.1016/S1002-0160(10)60050-1.

(5) Yuan, G.-L.; Qin, J.-X.; Lang, X.-X.; Li, J.; Wang, G.-H. Factors influencing the accumulation of organochlorine pesticides in the surface soil across the Central Tibetan Plateau, China. *Environmental Science: Processes & Impacts* **2014**, *16* (5), 1022-1028, 10.1039/C3EM00450C. DOI: 10.1039/C3EM00450C.

(6) Fu, S.; Chu, S.; Xu, X. Organochlorine pesticide residue in soils from Tibet, China. *BULLETIN OF ENVIRONMENTAL CONTAMINATION AND TOXICOLOGY* **2001**, *66* (2), 171-177. DOI: 10.1007/s001280000221.

(7) Tao, S.; Wang, W.; Liu, W.; Zuo, Q.; Wang, X.; Wang, R.; Wang, B.; Shen, G.; Yang, Y.; He, J.-s. Polycyclic aromatic hydrocarbons and organochlorine pesticides in surface soils from the Qinghai-Tibetan plateau. *Journal of Environmental Monitoring* **2011**, *13* (1), 175-181, 10.1039/C0EM00298D. DOI: 10.1039/C0EM00298D.

(8) Yang, R.; Zhang, S.; Li, A.; Jiang, G.; Jing, C. Altitudinal and Spatial Signature of Persistent Organic Pollutants in Soil, Lichen, Conifer Needles, and Bark of the Southeast Tibetan Plateau: Implications for Sources and Environmental Cycling. *Environmental Science & Technology* **2013**, *47* (22), 12736-12743. DOI: 10.1021/es403562x.

(9) Chen, D.; Liu, W.; Liu, X.; Westgate, J. N.; Wania, F. Cold-Trapping of Persistent Organic Pollutants in the Mountain Soils of Western Sichuan, China. *Environmental Science & Technology* **2008**, *42* (24), 9086-9091. DOI: 10.1021/es8018572.

(10) Chen, L.; Feng, Q.; He, Q.; Huang, Y.; Zhang, Y.; Jiang, G.; Zhao, W.; Gao, B.; Lin, K.; Xu, Z. Sources, atmospheric transport and deposition mechanism of organochlorine pesticides in soils of the Tibetan Plateau. *Science of The Total Environment* **2017**, *577*, 405-412. DOI: https://doi.org/10.1016/j.scitotenv.2016.10.227.

(11) Wang, C.; Wang, X.; Gong, P.; Yao, T. Residues, spatial distribution and risk assessment of DDTs and HCHs in agricultural soil and crops from the Tibetan Plateau. *Chemosphere* **2016**, *149*, 358-365. DOI: https://doi.org/10.1016/j.chemosphere.2016.01.120.

(12) Zhou, J.; Zhao, G.; Li, M.; Li, J.; Liang, X.; Yang, X.; Guo, J.; Wang, T.; Zhu, L. Three-dimensional spatial distribution of legacy and novel poly/perfluoroalkyl substances in the Tibetan Plateau soil: Implications for transport and sources. *Environment International* **2022**, *158*, 107007. DOI: https://doi.org/10.1016/j.envint.2021.107007.
(13) Zhu, N.; Schramm, K.-W.; Wang, T.; Henkelmann, B.; Fu, J.; Gao, Y.; Wang, Y.; Jiang, G. Lichen, moss and soil in resolving the occurrence of semi-volatile organic compounds on the southeastern Tibetan Plateau, China. *Science of The Total Environment* **2015**, *518-519*, 328-336. DOI: https://doi.org/10.1016/j.scitotenv.2015.03.024.
(14) Yuan, G.-L.; Xie, W.; Che, X.-C.; Han, P.; Liu, C.; Wang, G.-H. The fractional patterns of polybrominated diphenyl ethers in the soil of the central Tibetan Plateau, China: The influence of soil components. *Environmental Pollution* **2012**, *170*, 183-189. DOI: https://doi.org/10.1016/j.envpol.2012.07.011.
(15) Zhang, Z.; Xu, Y.; Wang, Y.; Li, Z.; Yang, C.; Rodgers, T. F. M.; Tan, F. Occurrence and distribution of organophosphate flame retardants in the typical soil profiles of the Tibetan Plateau, China. *Science of The Total Environment* **2022**, *807*, 150519. DOI: https://doi.org/10.1016/j.scitotenv.2021.150519.
(16) Liu, M.; Huang, L.; Li, X.; Liu, F.; Zhang, W.; Wang, Z.; Xu, Y.; Ke, R.; He, H.; Lou, Y. Occurrence and distribution of polyhalogenated carbazoles in eastern Tibetan Plateau soils along the slope of Mt. Qionglai. *Chemosphere* **2022**, *298*, 134200. DOI: https://doi.org/10.1016/j.chemosphere.2022.134200.
(17) Li, X.; Cao, L.; Lin, X.; Fu, W.; Gong, Z.; Zeng, X.; Fang, H.; Li, Z. Composition, Distribution, and Source Apportionment of Organochlorine Pesticides (OCPs) in Soil of a Chemical Industrial Park and its Surrounding in the Northeast of Qinghai-Tibet Plateau. *Water, Air, & Soil Pollution* **2023**, *234* (4), 236. DOI: 10.1007/s11270-023-06242-7.
(18) Tao, Y.-Q.; Lei, G.-L.; Xue, B.; Yao, S.-C.; Pu, Y.; Zhang, H.-C. Deposition and regional distribution of HCHs and p,p′ -DDX in the western and southern Tibetan Plateau: records from a lake sediment core and the surface soils. *Environmental Science and Pollution Research* **2014**, *21* (3), 1875-1883. DOI: 10.1007/s11356-013-2065-3.
(19) Wang, P.; Zhang, Q.; Wang, Y.; Wang, T.; Li, X.; Li, Y.; Ding, L.; Jiang, G. Altitude dependence of polychlorinated biphenyls (PCBs) and polybrominated diphenyl ethers (PBDEs) in surface soil from Tibetan Plateau, China. *Chemosphere* **2009**, *76* (11), 1498-1504. DOI: https://doi.org/10.1016/j.chemosphere.2009.06.045.
(20) Yuan, G.-L.; Sun, Y.; Li, J.; Han, P.; Wang, G.-H. Polychlorinated biphenyls in surface soils of the Central Tibetan Plateau: Altitudinal and chiral signatures. *Environmental Pollution* **2015**, *196*, 134-140. DOI: https://doi.org/10.1016/j.envpol.2014.10.006.
(21) You, J.; Chen, Z.-m.; Hou, X.-y.; Guo, J.-s.; Wang, C.-c.; Gao, J.-m. Occurrence, potential sources and risks of organophosphate esters in the high-elevation region, Tibet, China. *Science of The Total Environment* **2022**, *806*, 151348. DOI: https://doi.org/10.1016/j.scitotenv.2021.151348.
(22) Durant, J. L.; Leland, B. A.; Henry, D. R.; Nourse, J. G. Reoptimization of MDL

Keys for Use in Drug Discovery. *Journal of Chemical Information and Computer Sciences* **2002**, *42* (6), 1273-1280. DOI: 10.1021/ci010132r.
(23) Ghose, A. K.; Viswanadhan, V. N.; Wendoloski, J. J. Prediction of Hydrophobic (Lipophilic) Properties of Small Organic Molecules Using Fragmental Methods: An Analysis of ALOGP and CLOGP Methods. *The Journal of Physical Chemistry A* **1998**, *102* (21), 3762-3772. DOI: 10.1021/jp980230o.
(24) Wildman, S. A.; Crippen, G. M. Prediction of Physicochemical Parameters by Atomic Contributions. *Journal of Chemical Information and Computer Sciences* **1999**, *39* (5), 868-873. DOI: 10.1021/ci990307l.
(25) Labute, P. A widely applicable set of descriptors. *Journal of Molecular Graphics and Modelling* **2000**, *18* (4), 464-477. DOI: https://doi.org/10.1016/S1093-3263(00)00068-1.
(26) Todeschini, R.; Vighi, M.; Finizio, A.; Gramatica, P. 30-Modelling and Prediction by WHIM Descriptors. Part 8. Toxicity and Physico-chemical Properties of Environmental Priority Chemicals by 2D-TI and 3D-WHIM Descriptors. *SAR and QSAR in Environmental Research* **1997**, *7* (1-4), 173-193. DOI: 10.1080/10629369708039130.
